# P-2272. The CARV challenge: How do hematological malignancy patients struggle with diverse CARV pathogens

**DOI:** 10.1093/ofid/ofae631.2425

**Published:** 2025-01-29

**Authors:** Jon Salmanton-Garcia, Francesco Marchesi, Jorge Labrador, Nicola Fracchiolla, Yavuz M Bilgin, Maria Ilaria Del Principe, Ľuboš Drgoňa, Laman Rahimli, Gaëtan Plantefève, Jens Van Praet, Nicola Sgherza, Klara Piukovics, Ildefonso Espigado, Inmaculada Heras Fernando, Oliver A Cornely, Livio Pagano

**Affiliations:** University Hospital Cologne, Cologne, Germany, Cologne, Nordrhein-Westfalen, Germany; IRCCS Regina Elena National Cancer Institute, Rome, Lazio, Italy; Hospital Universitario de Burgos, Burgos, Castilla y Leon, Spain; Fondazione IRCCS Ca' Granda-Ospedale Maggiore Policlinico di Milano, Milan, Lombardia, Italy; ADRZ, Goes, Zeeland, Netherlands; Università degli studi di Roma "Tor Vergata", Rome, Lazio, Italy; Comenius University and National Cancer Institute, Bratislava, Bratislava, Slovakia; University Hospital Cologne, Cologne, Nordrhein-Westfalen, Germany; Centre Hospitalier d'Argenteuil, Argenteuil, Ile-de-France, France; AZ Sint-Jan Brugge-Oostende AV, Brugge, West-Vlaanderen, Belgium; Policlinico di Bari, Bari, Puglia, Italy; University of Szeged, Szeged, Csongrad, Hungary; University Hospital Virgen del Rocio, Seville, Andalucia, Spain; Hospital Morales Messeguer, Seville, Andalucia, Spain; University of Cologne, Faculty of Medicine and University Hospital Cologne, Cologne, Nordrhein-Westfalen, Germany; Fondazione Policlinico Universitario Agostino Gemelli - IRCCS, Rome, Lazio, Italy

## Abstract

**Background:**

With growing concerns about the effects of community-acquired respiratory viruses (CARVs) on hematological malignancy patients, proactive measures are necessary to minimize risks and improve treatment results. This study aimed to collect and analyze epidemiological, management, and outcome data from these patients with CARV infections to develop customized clinical management approaches.

**Table 1.**
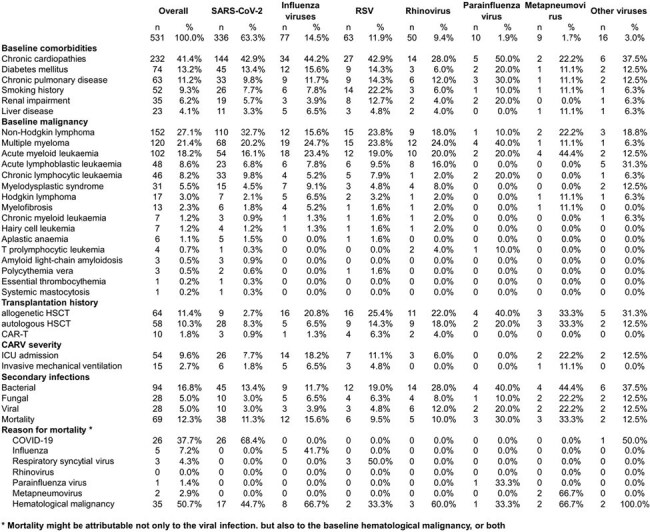
Patient characterisitics

**Methods:**

Utilizing an online registry, data from of CARV infections in hematological malignancy patients were collected from January 2023 to January 2024, spanning 53 sites across 21 countries.

**Results:**

Our survey included 561 cases of CARV in hematological malignancy patients, with a majority from Italy (39%) and Spain (19%). Severe acute respiratory syndrome coronavirus 2 (SARS-CoV-2) made up 63% of cases, followed by influenza (15%), respiratory syncytial virus (12%), and rhinovirus (9%). The distribution of CARVs mirrored the overall prevalence across different malignancies, with 27% in acute leukemias and non-Hodgkin lymphoma each, and 21% in multiple myeloma. Stem cell transplantation or CAR-T therapy preceded CARV infection in 24% of patients, particularly with metapneumovirus (67%) and parainfluenza (60%). Chronic cardiopathies were the most common comorbidity (41%). Critical illness was more prevalent in metapneumovirus (22%) and influenza cases (18%) compared to RSV (11%) or SARS-CoV-2 (8%). Metapneumovirus and parainfluenza had the highest mortality rates (33% and 30%, respectively), surpassing those of influenza (16%) or SARS-CoV-2 (11%). SARS-CoV-2 (68%) and RSV (50%) had the highest associated mortality rates. Progression of baseline malignancy contributed to 50% of overall mortalities. Further details are provided in Table 1.

**Conclusion:**

Our study underscores the significant impact of CARV on hematological malignancy patients, especially with SARS-CoV-2 predominance. The distribution of CARVs reflected malignancy prevalence, with acute leukemias and non-Hodgkin lymphoma most affected. Moreover, we found heightened severity and mortality rates associated with specific CARV pathogens such as metapneumovirus and parainfluenza.

**Disclosures:**

Oliver A. Cornely, Prof. Dr., Abbott: Honoraria|Abbvie: Advisor/Consultant|Abbvie: Honoraria|AiCuris: Advisor/Consultant|Akademie fur Infektionmedizin: Honoraria|Al-Jazeera Pharmaceuticals/Hikma: Honoraria|amedes: Honoraria|AstraZeneca: Honoraria|Basilea: Advisor/Consultant|Biocon: Advisor/Consultant|BMBF: Grant/Research Support|Boston Strategic Partners: Advisor/Consultant|CIdara: Advisor/Consultant|CIdara: Expert Testimony|CIdara: Grant/Research Support|CIdara: Participation on a DRC or DSMB|CoRe Consulting: Stocks/Bonds (Private Company)|Deutscher Arzteverlag: Honoraria|DZIF: Grant/Research Support|EasyRadiology: Stocks/Bonds (Private Company)|EU-DG RTD: Grant/Research Support|F2G: Grant/Research Support|Gilead: Advisor/Consultant|Gilead: Grant/Research Support|Gilead: Honoraria|Grupo Biotoscana/United Medical/Knight: Honoraria|GSK: Advisor/Consultant|GSK: Honoraria|IQVIA: Advisor/Consultant|IQVIA: Participation on a DRC or DSMB|Janssen: Advisor/Consultant|Janssen: Participation on a DRC or DSMB|Matinas: Advisor/Consultant|MedPace: Advisor/Consultant|MedPace: Grant/Research Support|MedPace: Participation on a DRC or DSMB|Medscape/WebMD: Honoraria|MedUpdate: Honoraria|Menarini: Advisor/Consultant|Moderna: Honoraria|Molecular Partners: Advisor/Consultant|MSD: Grant/Research Support|MSD: Honoraria|MSG-ERC: Advisor/Consultant|Mundipharma: Advisor/Consultant|Mundipharma: Grant/Research Support|Mundipharma: Honoraria|Noscendo: Honoraria|Noxxon: Advisor/Consultant|Octapharma: Advisor/Consultant|Octapharma: Grant/Research Support|Pardes: Advisor/Consultant|Partner Therapeutics: Advisor/Consultant|Patent: US18/562644|Paul-Martini-Stiftung: Honoraria|Pfizer: Advisor/Consultant|Pfizer: Grant/Research Support|Pfizer: Honoraria|PSI: Advisor/Consultant|PSI: Participation on a DRC or DSMB|Pulmocide: Participation on a DRC or DSMB|Sandoz: Honoraria|Scynexis: Advisor/Consultant|Scynexis: Grant/Research Support|Seqirus: Advisor/Consultant|Seqirus: Honoraria|Seres: Advisor/Consultant|Shionogi: Advisor/Consultant|Shionogi: Honoraria|streamedup!: Honoraria|The Prime Meridian Group: Advisor/Consultant|Touch Independent: Honoraria|Vitis: Honoraria

